# A Treatise on the Science and Practice of Midwifery

**Published:** 1885-08

**Authors:** 


**A Treatise on the Science and Practice of Midwifery.** By W. L. PLAYFAIR, M. D., F. R. C. P. Fourth American, from the fifth English, edition. With notes and additions by ROBERT P. HARRIS, M. D. With three plates and 201 illustrations. Philadelphia: Lea Brothers & Co. 1885.

This excellent work needs no commendation from our pen. For many years, it has maintained a deservedly high reputation among teachers as a text-book, and in the profession as a guide to the practical experiences which attend the obstetrician. The present edition, under the supervision of Dr. Harris, has been carefully revised, and many portions of the work re-written. The chapter on conception and generation has been made to include the more recent advances in embryology, and the work has been adapted to the wants and circumstances of this continent. Among medical students, the work is a most valuable text-book, and we have had occasion to recommend it to our classes. To the general practitioner, it is an equally valuable work.

